# Expanding the reach of vaccinology training in Africa: leveraging the success of the Annual African Vaccinology Course

**DOI:** 10.3389/frhs.2023.1119858

**Published:** 2023-09-01

**Authors:** Nayna Manga, Edina Amponsah-Dacosta, Gregory Hussey, Rudzani Muloiwa, Benjamin M. Kagina

**Affiliations:** ^1^ Vaccines for Africa Initiative, School of Public Health and Family Medicine, Faculty of Health Sciences, University of Cape Town, Cape Town, South Africa; ^2^ Department of Pediatrics and Child Health, Red Cross War Memorial Children’s Hospital, Faculty of Health Sciences, University of Cape Town, Cape Town, South Africa

**Keywords:** vaccines, vaccinology, health education, online training, COVID-19, Africa

## Abstract

**Introduction:**

It is estimated that one in five African children lack access to recommended life-saving vaccines. This situation has been exacerbated by the COVID-19 pandemic which disrupted routine immunization services in several parts of the region. To better support recovery efforts and get immunization programmes back on track, policy makers, programme managers, immunization providers and academics need continuous upskilling. Unfortunately, the vaccinology training needed by these cadres remains limited and oftentimes inaccessible within our context. In addition, cadres should be continuously updated on advances in vaccinology so as to keep abreast with this rapidly evolving field. This calls for new and accessible approaches to training vaccinologists in Africa where the demand is high.

**Methods:**

The aim of this proof-of-concept study was to ascertain the training needs of alumni of the Annual African Vaccinology Course and assess the effectiveness of an online webinar series in meeting those needs.

**Results:**

We found that alumni from across Africa required refresher training to gain up-to-date information about new developments in vaccinology, leverage opportunities to reinforce and consolidate their knowledge, and exchange country-specific experiences with their counterparts. A prominent motivation for refresher training was the rapid developments and challenges brought on by the COVID-19 pandemic. Drawing on the expressed needs of our alumni, we developed a webinar training series. This series aimed to provide participants with training on current and emerging trends in vaccinology with a focus on the regional context. Online participation in the webinar series was found to be comparable to previous in-person training, reaching a diverse group of cadres, and allowing for participation of a richer global faculty due to fewer cost constraints. Further to this, a post-training survey indicated that generally, alumni training needs were successfully met.

**Discussion:**

The findings suggest that an online approach can be used to expand the reach of vaccinology training in Africa.

## 1. Introduction

The Annual African Vaccinology Course (AAVC) is a five-day in-person course which was developed in 2005 by the Vaccines for Africa Initiative (VACFA) based at the University of Cape Town in South Africa, in response to the growing demand for vaccinology training in Africa (1, 2). Each year since then, an average of 60 participants comprising of members of National Immunization Technical Advisory Groups (NITAGs), immunization programme managers from national, sub-national and district levels, immunization providers from both private and public health sectors, scientists, postgraduate students and postdoctoral research fellows, as well as individuals working with non-governmental or not-for-profit agencies, and the pharmaceutical industry, have been trained through the AAVC. Notably, this has amounted to an alumni pool of 992 individuals from 44 of the 54 African countries who have received vaccinology training through the AAVC between 2005 and 2020 (1, 2). The broader goal of the AAVC is to empower African vaccinologists to be directly involved in the design and implementation of home-grown solutions to the local challenges facing immunization programmes across the continent.

Despite the remarkable efforts of the AAVC and other training initiatives elsewhere, a 2019 landscape analysis of vaccinology research and training in sub-Saharan Africa found that training opportunities for vaccinologists in the region remains limited (3). This study identified only ten vaccinology courses, including the AAVC. The courses identified vary in duration, content and level depending on the target audience, and are mainly delivered through an in-person format with fewer opportunities for distance or remote learning (3). Evidently, there is a need for expanding vaccinology training opportunities in Africa, while simultaneously adapting and synergizing existing efforts in order to meet the evolving needs of vaccinologists and the immunization systems they work in (3, 4).

In addition to the limited availability of vaccinology education for first-time trainees, Duclos et al. (5), report that alumni (vaccinologists who have received training previously) encounter challenges with keeping their knowledge updated due to time, availability, and financial constraints. In addition to this, very few vaccinology courses offer refresher training or support for their alumni. Ensuring that alumni remain engaged and continue their training in vaccinology has become crucial due to the introduction of new vaccine technologies over time, the resurgence of previously controlled vaccine preventable diseases (VPDs), the emergence of new VPDs like COVID-19, and growing needs for countering vaccine mis- and dis-information. Empowering vaccinologists with up-to-date knowledge ensures that immunization programmes are appropriately and competently administered and monitored (5, 6). This can be achieved by providing alumni with refresher vaccinology training through online approaches such as massive open online courses, permanent access to online repositories with continuously updated training resources, and regular meetings or workshops delivered through webinar formats.

In 2020, the 16th edition of the AAVC was delivered in a hybrid format for the very first time, allowing for limited in-person attendance (*n* = 34) and complemented by online participation (*n* = 54) due to COVID-19 restrictions on social gatherings that year (2). This hybrid format allowed for a larger number of participants to attend this popular course, prompting the AAVC conveners to start exploring online vaccinology training as a strategy to make vaccinology training more accessible on the continent. Further to this, conveners noted a trend of an increasing number of AAVC alumni applying to re-attend the course. In response to this, the conveners of the AAVC sought to develop a refresher vaccinology training course as an avenue to keep its alumni updated with new developments in the field and to foster opportunities for meaningful engagement and collaboration. This study aimed to (a) ascertain the vaccinology refresher training needs of AAVC alumni; (b) develop a cost-effective and widely accessible refresher vaccinology webinar-based course tailored to the African context; and (c) provide proof-of-concept evidence by investigating the success of this training as perceived by participants.

## 2. Methods

### 2.1. Assessing the vaccinology training needs of AAVC alumni

A questionnaire was devised to survey AAVC alumni about the need for a refresher training course. This questionnaire was reviewed and piloted among AAVC organizing committee and faculty members who were also alumni but were not selected as part of the participants for this study. The study population consisted of alumni who attended the AAVC in 2011 and 2013 to 2020. Cohorts who attended from 2005 to 2010 were excluded because many of their contact details were outdated. The 2012 cohort was excluded because the 2012 AAVC was held back-to-back with the First International African Vaccinology Conference with some participants attending both events (7). A Google form survey consisting of 8 closed-ended and 2 open-ended questions (Supplementary File S1) was devised to ascertain the vaccinology refresher training needs of AAVC alumni. An invitation with a link to the survey was emailed to each alumnus on 9 February 2022. Data from the responses received by 20 February 2023 were entered into a Microsoft Excel® (Version 2205 Build 16.0.15225.20368) form designed for this study and analyzed independently by two researchers (NM and EA-D) using inductive descriptive analysis to identify thematic categories.

### 2.2. Refresher vaccinology training webinar series

Drawing on the findings of the survey, we developed a five-part vaccinology training programme tailored to the expressed needs of alumni and delivered via a webinar format on Zoom—an innovative video conferencing platform—between 22 April and 27 May 2022. A copy of the detailed programme for this webinar series is provided in Supplementary File S2. All participants were requested to register for each webinar via the VACFA website (www.vacfa.uct.ac.za), indicating the year they last attended the AAVC, their current occupation or role, email address and whether they wanted to be added to an AAVC alumni email group. While the webinar series was targeted at AAVC alumni, it was not restricted to them and was made available to anyone who would benefit. As such the programme was distributed via email among AAVC alumni, as well as the broader VACFA network, and uploaded onto the VACFA website. Registered participants were provided a link to each webinar in the series. The registration and attendee data were entered in Microsoft Excel® (Version 2205 Build 16.0.15225.20368) for basic descriptive analysis.

### 2.3. Post-training survey and feedback assessment

To determine whether the objectives of the webinar series were met, participants were requested to provide feedback via email after each webinar. Unfortunately, the response rate was dismal. Therefore, a post-training questionnaire was devised, and the registrants sent an email containing a link to the survey on Google Forms (Supplementary File S3). Participants were requested to complete the survey from 6 to 14 June 2022. The data from the survey responses were then entered into Microsoft Excel® (Version 2205 Build 16.0.15225.20368) and analyzed using inductive descriptive analysis to identify thematic categories.

### 2.4. Ethical considerations

Although this study involved human participants, it is categorized as a quality improvement audit of educational interventions, thus formal approval from an Institutional Ethics Committee was not required. In addition, written informed consent was not required in order to participate in this study in accordance with the national legislation and the institutional requirements.

## 3. Results

### 3.1. Outcomes of the alumni training needs assessment survey

#### 3.1.1. Survey response rate

A total of 547 alumni from our 2011 and 2013–2020 AAVC cohorts for whom email addresses were available were invited to complete the online survey. Of these, 101 (18.5%) survey responses were received. One duplicate response was detected by cross-referencing email addresses with responses and thus was not included in the final analysis. Therefore, 100 unique responses were analyzed which equates to an 18.3% (100/547) response rate.

Six respondents indicated that they had attended the course more than once, five had attended twice and one attended five times. Table 1 shows the responses by cohort based on the year of attending the AAVC. Most of the responses were received from those who attended the recent editions of the course; 23% (17/74) in 2017, 26.7% (16/60) in 2019, and 25.6% (20/78) in 2020. Fewer responses were received from alumni who attended earlier offerings of the AAVC.

**Table 1:** Response rate for the AAVC alumni training needs survey per participating year.

AAVC Cohort^a^	Number of alumni surveyed	Number of responses received	Response rate
2011	58	7	12.1%
2013	50	6	12.0%
2014	63	7	11.1%
2015	54	11	20.4%
2016	50	7	14.0%
2017	74	16	21.6%
2018	60	10	16.7%
2019	60	16	26.7%
2020	78	20	25.6%
Total	**547**	**100**	**18**.**3%**

^a^ Participants who indicated attending the AAVC more than once were assigned to the first year they attended the course.

#### 3.1.2. Vaccinology refresher training needs of AAVC alumni

**Figure 1:**
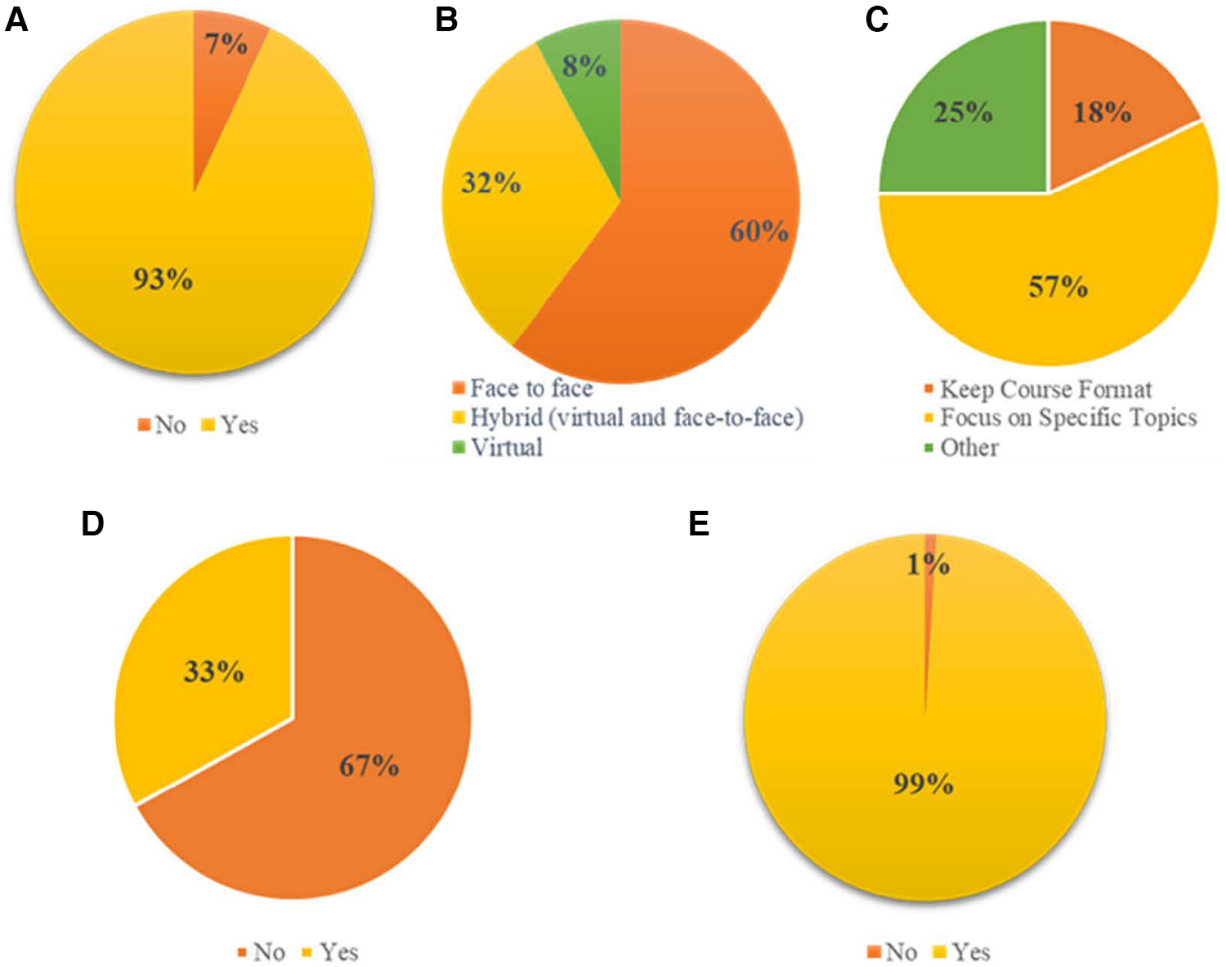
Distribution of responses to the alumni training needs assessment survey. (**A**) The need for a refresher in vaccinology training. (**B**) Course delivery preference. (**C**) Course organisation. (**D**) Attendance of other vaccinology course after AAVC. (**E**) Willingness to attend online vaccinology course as refresher training.

Of all respondents, 93% (93/100) answered “yes” when asked “Do you think there is a need for refresher training?” (Figure 1A). Of the seven respondents who did not see the need for a refresher, six opted not to provide reasons for their response. However, one respondent did state that: “*I think people should be given access to online modules. Anyone who needs a refresher should take the online course*” [AAVC 2018 alumnus]. Three main themes emerged from the reasons given by respondents who indicated needing a refresher course.

##### 3.1.2.1. Theme 1: need to obtain up-to-date training on new developments in vaccinology

Firstly, 80% (80/100) of the respondents indicated that they wanted to attend the refresher in order to obtain up-to-date information about new developments in vaccinology, including concepts related to policies, immunization programmes, vaccines, vaccination trials and technology. For example, one of the respondents stated: “*So many things have changed in the immunization landscape since we last attended the course*” [AAVC 2016 alumnus].

A prominent motivation for the need to be updated was the rapid developments and challenges brought on by the COVID-19 pandemic. Alumni wanted to learn about the development of new COVID-19 vaccines and the implementation and roll-out of COVID-19 vaccination programmes in Africa: “*Especially with Covid-19 there has [sic] been advances in the vaccine field that would be most helpful to learn of*” [AAVC 2017 alumnus] and “*Vaccinology is a rapidly changing field especially with the emergence of COVID-19*” [AAVC 2017 alumnus]. They were also interested in learning about managing immunization programmes during the pandemic in the context of lockdown restrictions: “*To have new ideas on how to implement during Covid-19 Pandemic*” [AAVC 2019 alumnus] and “*the context of the Covid19 pandemic requires a new way of managing vaccines*” [AAVC 2013 alumnus].

The need for refresher training was also due to concerns about emerging diseases that would require vaccination: “*New pathogens e.g., COVID -19 (Emerging)*” [AAVC 2011 alumnus], and “*The occurrence of new diseases like Ebola and corona, the vaccine knowledge to the community is very much needed*” [AAVC 2014 alumnus]. Information was also needed about issues which were becoming more prominent and concerning during the COVID-19 pandemic, such as vaccine hesitancy and the consequent need for greater advocacy: “*I need knowledge on vaccine hesitancy (vaccine preventable diseases) and the emergence of new diseases (COVID-19) and what needs to be done to curtail it especially in [a] country like mine*” [AAVC 2019 alumnus], “*News [sic] challenges on immunisation field: infodemics, hesitancy, anti-vax*” [AAVC 2011 alumnus] and “*vaccines [sic] hesitancy knowledge to the community*” [AAVC 2019 alumnus].

##### 3.1.2.2. Theme 2: need to reinforce and consolidate knowledge in vaccinology

Another prominent theme identified among 22% (22/100) of respondents was the anticipated benefits of attending refresher training. Alumni expressed that a refresher course would give them the opportunity to reinforce and consolidate their knowledge. Here, it was considered that a refresher course would be of benefit to those attending as they had gained more experience since they last attended the course and could glean more from the refresher:

*“Some of [us] attended the course in our early carrier [sic] stage, when not many things were fully understood and appreciated. With more experience in the field of Immunisation and vaccination, the course will be more helpful”* [AAVC 2014 alumnus]

and,

*“Participation in the NITAG [National Immunization Technical Advisory Group] makes me see the relevance; I could benefit more now”* [AAVC 2018 alumnus].

In addition, the need for obtaining reliable scientific evidence was raised as shown by one response:

*“COVID-19 Pandemic alongside with it vaccine production brought about conflicting Scientific data and information perspectives. Attending a new vaccinology workshop session may assist us [to] have a clear scientific perspective on COVID-19 vaccine development, and booster information. Moreover we may refresh our skills in vaccine development in general”* [AAVC 2015 alumnus].

Alumni thought that a refresher course would help bring them up to date with new developments and thereby strengthen their capacity to execute their roles:

*“I work as a stock control pharmacist at a wholesaler and have to have most answers when my clients ask me about vaccines that I store. The course [AAVC in 2017] helped to improve how we manage our inventory and lastly helped with distribution patterns to our clients that is maintaining of temperature from our stores to end users”* [AAVC 2017 alumnus].

##### 3.1.2.3. Theme 3: need for networking and knowledge exchange

The final emergent theme was related to the perceived opportunity for networking and strengthening of collaborations among African vaccinologists which was expressed by 32% (32/100) of respondents: “*refresh networks; share information*” [AAVC 2019 alumnus], “*To share knowledge and network with other African vaccinologists*” [AAVC 2011 alumnus], and “*Strengthen network with other vaccinologists in Africa*” [AAVC 2017 alumnus].

When it came to alumni's preference for the delivery of refresher training (Figure 1B), 60% (60/100) indicated that they preferred a face-to-face mode of delivery, 32% (32/100) preferred a hybrid format, while only 8% (8/100) preferred a virtual mode of delivery. Further to this, when alumni were asked how the course conveners could best meet their needs, 57% (57/100) preferred that the refresher be focussed on specific topics determined by both alumni and convenors (Figure 1C). In ascertaining whether alumni had sought additional training since attending the AAVC, we found that 67% (67/100) had not attended another vaccinology course (Figure 1D). Alumni were then informed that AAVC conveners had partnered with other global vaccinology course conveners to meet the growing demand for vaccinology training in Africa as well as other regions and were asked if as an alternative to returning to the 5-day, in-person AAVC format for refresher training, they would be open to attend any other online vaccinology course as a strategy for refresher training. The overwhelming majority, 99% (99/100), were in favour of this option (Figure 1E).

### 3.2. Development and uptake of the refresher vaccinology training webinar series

The overarching aim of the webinar series was to address the training needs of the AAVC alumni as ascertained in the survey. In this regard, the primary objectives of the webinar series were to, (i) provide participants with up-to-date information about new developments in vaccinology, (ii) reinforce and consolidate participants' knowledge, (iii) provide an opportunity for networking and strengthening collaborations especially in the African context, and (iv) broaden the understanding of the challenges and opportunities in vaccinology at regional and global levels.

The series consisted of five webinars held via Zoom. All presentations were delivered in English. Generally, the format of the webinars was 3–4 keynote presentations followed by a moderated discussion for 50 min. The webinar series covered topics such as the Immunization Agenda 2030, basic principles of immunology, history and rationale of vaccination schedules, developing vaccines for pandemic preparedness, surveillance of VPDs and other infectious diseases, and the application of human centred design principles in vaccinology. Key action points for the Immunization Agenda 2030 focussed on successes and challenges of National Immunization Programmes (NIPs) in Africa. The impact of the COVID-19 pandemic was discussed in the context of NIPs in Africa. Emerging trends in immunology and vaccinology were also discussed in addition to the vaccine manufacturing capacity for Africa. Issues around generating demand for vaccination to increase vaccine confidence were also addressed. A Human Centred Design (HCD) consultant facilitated the final webinar on the application of HCD in improving access and acceptability of immunization services (Supplementary File S2). This HCD session was designed to expose participants to tools they can use to foster innovations in addressing key immunization challenges unique to the African context.

Table 2 provides a summary of the number of registrants, and attendees. Unfortunately, registration and attendee data for the third webinar was lost due to changes to the online registration platform. On average, 86.3% (272/315) of registrants were AAVC alumni and 52 individuals attended each webinar in the series. Overall, 176 individuals attended at least one webinar and participants were from 40 different African countries.

**Table 2:** Registration and uptake of the refresher vaccinology training webinar series.

Webinar series no.	Number of registrations	Number of registrants who were AAVC alumni^b^ (%)	Number of attendees^b^ (%)	Number of African countries represented
1	80	71 (88.8%)	64 (80%)	30
2	73	67 (91.8%)	49 (67.1%)	22
3^a^	—	—	—	—
4	104	86 (82.7%)	61 (58.7%)	28
5	58	48 (82.8%)	33 (56.9%)	22

^a^ Data could not be retrieved for webinar #3 due to changes in the online registration platform.

^b^ The denominator is the number of registrations per webinar.

A diverse pool of 16 local, regional, and international faculty members delivered talks and moderated discussions during the webinar series. Faculty members were representatives from academia, global health agencies, non-governmental organizations, and the vaccine industry.

### 3.3. Feedback from the post-training survey

**Figure 2:**
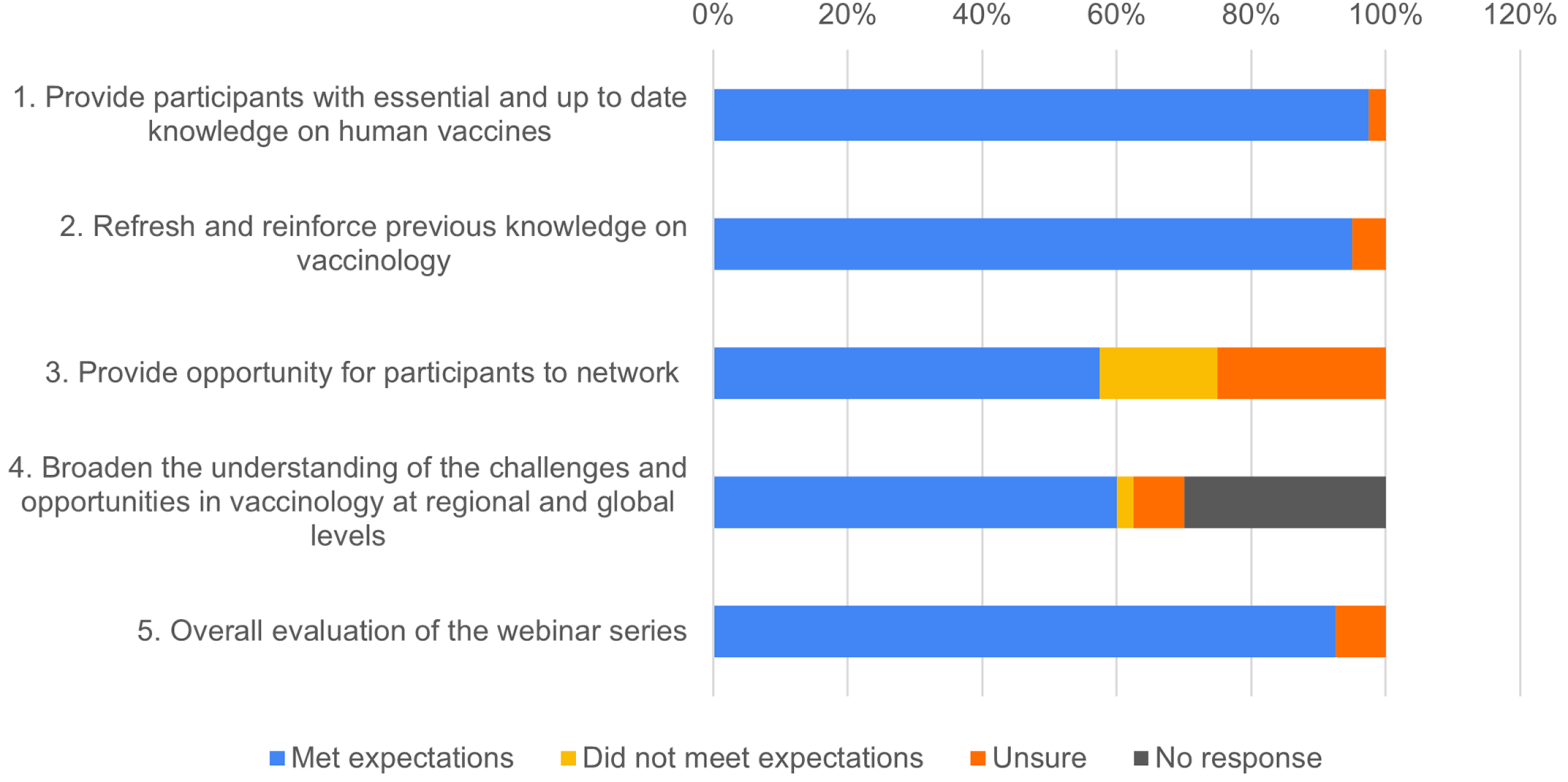
AAVC alumni feedback on the refresher vaccinology training webinar series.

Of the 176 individuals who attended at least one webinar during the five-part series, 22.7% (40/176) responded to the post-webinar series survey. Eighty percent (32/40) of those surveyed attended two or more webinars in the series while 18% (7/40) attended all five webinars. When asked the extent to which the webinar series met its first two objectives, 97.5% (39/40) and 95% (38/40) of respondents respectively, indicated that their need for (1) essential and up to date knowledge on human vaccines, and (2) reinforcing previous knowledge on vaccinology was successfully met (Figure 2). In relation to networking opportunities, 57.5% (23/40) of respondents indicated that the webinar series met their expectations while 25% (10/40) and 17.5% (7/40) were unsure or did not agree that this objective was met, respectively. Sixty percent (24/40) expressed that the webinar series broadened their understanding of the challenges and opportunities in vaccinology from both regional and global perspectives (Figure 2). Finally, when asked about their overall evaluation of the webinar series, 93% (37/40) indicated that their expectations had been met.

#### 3.3.1. Theme 1: need for inclusive training formats

Alumni expressed that the refresher vaccinology training webinar series was a good initiative and wanted it to be rolled out regularly with broadened participation including making the webinars more accessible to other participants as well as making presentations available to those unable to attend due to work commitments or other constraints. While some respondents stated that the online format allowed more people to attend, one respondent raised the challenge of internet access: “*what are the initiatives put in place to accommodate potential participants from some countries without internet connection or access?*” [Respondent #21]. Another expressed the need to make the training more inclusive by providing language translations: “*we would like this course to be translated into other languages such as French and Portuguese because this will facilitate deep understanding and good assimilation of the courses*” [Respondent #11].

Additional feedback related to the format or structure of the webinars which in some instances interfered with work commitments: “*please avoid long sessions IN a day, one that goes longer than 2 h becomes difficult to actively participate in due to work and other commitments*” [Respondent #24].

#### 3.3.2. Theme 2: need for training environments that foster meaningful collaboration

One respondent suggested that future webinars provide more opportunities to network and establish collaborations: “*I think the coming webinar should increase the environment for researchers to network and establish collaborations*” [Respondent #35]. This gap in opportunities to actively network emerged as a key theme among alumni who participated in the webinar series. To help bridge this gap, alumni were asked if they wanted to be part of an AAVC alumni group that VACFA was establishing. In response to this, 94% (167/176) of the registrants indicated that they would like to be part of the group. The contact details of those who responded affirmatively were collated for the formation of a mailing list. This group was also invited to access a resource repository on the VACFA website containing e-resources which have been assembled to supplement the webinar series.

## 4. Discussion

In 2018, the World Health Organization reported that one in five African children still lack access to recommended life-saving vaccines (8). Since then, immunization gaps within the African region have been amplified following disruptions to routine services brought on by the COVID-19 pandemic (9, 10). A capable health workforce will be an integral health systems resource for recovery efforts aimed at getting immunization programmes back on track. It is not surprising then that the primary motivation for refresher vaccinology training among alumni of our AAVC was the COVID-19 pandemic and its influence on the rapid developments within the immunization landscape. In addition, alumni sought opportunities to reinforce and consolidate their knowledge having had further experience in the field since they last attended the AAVC, as well as an environment that fostered networking and collaboration with their counterparts across the continent. In response to this, we developed a refresher vaccinology training series, delivered in a webinar format to enhance the accessibility of the course. The findings from our post-training survey indicate that overall, the webinar training series was an overwhelming success, having met the expectations of 93% of alumni surveyed.

At a 2018 workshop, leaders of 26 advanced vaccinology courses conducted an extensive review of vaccinology courses available globally (5). One of the conclusions of this review was that there was a need to facilitate post-course cascade training for alumni. Further to this, online approaches to adult education were proposed as efficient and cost-effective strategies for providing accessible refresher training. Such an approach would also negate the necessity for participants to attend multiple courses or to attend the same course again, thus allowing for a greater number of individuals to have access to vaccinology training (5). It is with this in mind that we sought to build on the success of the AAVC which has been at the forefront of vaccinology training in Africa for the past 16 years, amassing an alumni pool of 992 NITAG members, immunization programme managers and providers, academics, and individuals from relevant non-governmental or not-for-profit agencies, and the pharmaceutical industry (1, 2). Generally, the reach of the refresher webinar training series was found to be comparable to the in-person format of the AAVC. Conveners were able to leverage the faculty of the AAVC, with the added benefit of including a richer and more internationally representative faculty membership without the travel and logistics cost limitations imposed by in-person training.

Two key themes emerged from the post-webinar training survey, the first being the need for inclusive refresher vaccinology training webinar series. While the webinar format may allow for wider participation, settings with unstable internet access may not be conducive for online training. To mitigate this challenge, we created a repository accessible via the VACFA website where recordings and slide presentations were made available soon after the series to broaden the reach of the training materials for those who were unable to attend or access the live webinars. Analysis of web traffic on this site going forward will give an indication of the reach and usefulness of this information to AAVC alumni. Translation of the webinars into French and Portuguese would also make the information more accessible to a greater African audience. The second theme was on the need for an environment that fosters meaningful collaboration among African vaccinologists. This emerged as a critical limitation of the webinar format. Future refresher training initiatives will have to explore the usefulness of discussion groups or breakout rooms as an avenue for further interaction and improved engagement among alumni.

The findings of this study should be interpreted with careful consideration of some limitations. First, the response rates of the needs assessment and the post-webinar surveys were sub-optimal. While we attempted to survey our large pool of alumni, deactivated or unmonitored email addresses meant that we could not reach most of them. With the establishment of alumni groups and mailing lists, it is anticipated that contact details will be maintained and regularly updated. While findings may be unique to alumni from the African context, this study does provide significant lessons for guiding other training initiatives intending to implement continuous vaccinology training.

## 5. Conclusion

Addressing the gaps and growing demand for vaccinology training in Africa relative to the rest of the world requires more than the provision of once-off training. Alumni of vaccinology courses require regular upskilling in order to strengthen their capacity to execute their roles and make lifesaving vaccines accessible to those who need them the most. This study supports the use of an online approach for providing cadres working in the immunization space with continuous vaccinology training. The findings suggest that online training is a practical and cost-effective approach to expanding vaccinology expertise in Africa.

## Data Availability

The datasets presented in this study can be found in online repositories. The names of the repositories can be found in the article/**Supplementary Material**.
