# Supplementary material for: Expanding the reach of vaccinology training in Africa: leveraging the success of the Annual African Vaccinology Course
Source: Front Health Serv. 2023 Sep 1;3:1119858. doi: 10.3389/frhs.2023.1119858 (PMC10505672; doi:10.3389/frhs.2023.1119858)
Supplement: Supplementary file 1 [file Datasheet1.pdf]

AAVC Survey 2022

Questionnaire to understand the reasons behind vaccinology course alumni wanting to return for the same vaccinology course.

Introduction and rationale:
Greetings from the VACFA's Annual African Vaccinology Course (AAVC) conveners. We have sent you this short questionnaire because you are a AAVC alumni. We have noticed an increasing trend of past alumni applying to attend the 5-day AAVC. We would like to understand the reasons why some of you would like to attend AAVC again. We have prepared 7 questions that will take you about 10 minutes to complete. Your answers to these questions will give the AAVC conveners and partners an insight on the issue. Kindly complete and submit by 20 February 2022. Thank you.
If you have any questions, please contact Dr. Benjamin Kagina (Benjamin.kagina@uct.ac.za)

*Required

1. Email *

2. 1) Which year/s did you attend the AAVC (Multiple selection permitted) *

Tick all that apply.

- [ ] 2005
- [ ] 2006
- [ ] 2007
- [ ] 2008
- [ ] 2009
- [ ] 2010
- [ ] 2011
- [ ] 2012
- [ ] 2013
- [ ] 2014
- [ ] 2015
- [ ] 2016
- [ ] 2017
- [ ] 2018
- [ ] 2019
- [ ] 2020

3. 2) Do you think that there is a need of refresher training? *

Mark only one oval.

- Yes
- No

4. 3) Can you tell us the THREE MAIN reasons why you would like to apply to attend the refresher training?

5. 4) If given the opportunity to attend a refresher course, tell us how the conveners/ organizers can best meet your needs in terms of course organization *

Mark only one oval.

- a. Keep the course format the same as was the last time you attended
- b. Focused on specific topics that are decided by alumni and the conveners
- Other:

6. 5) Can you please tell us your preference for attending the refresher training? *

Mark only one oval.

- Virtual
- Face to face
- Hybrid (virtual and face-to-face)

7. 6) Have you ever attended any other vaccinology course (online or face-to-face) after you attended the AAVC? *

Mark only one oval.

- Yes
- No

8. 6a) If yes, name of the course and the year you attended

9. 7) AAVC conveners have partnered with other global vaccinology course conveners to meet the growing demand for vaccinology training in Africa/ other regions. As an alternative to returning to AAVC for refresher training, would you be open to attend any other online vaccinology course as a strategy for refresher training? *

Mark only one oval.

- Yes
- No

10. Any general comments that you would like to share with AAVC convenors?

This content is neither created nor endorsed by Google.

Google Forms
