# Supplementary material for: Expanding the reach of vaccinology training in Africa: leveraging the success of the Annual African Vaccinology Course
Source: Front Health Serv. 2023 Sep 1;3:1119858. doi: 10.3389/frhs.2023.1119858 (PMC10505672; doi:10.3389/frhs.2023.1119858)
Supplement: Supplementary file 2 [file Datasheet2.pdf]

UNIVERSITY OF CAPE TOWN
IYUNIVESITHI YASEKAPA • UNIVERSITEIT VAN KAAPSTAD

VACFA vaccines for africa

Vaccines for Africa Initiative
Faculty of Health Sciences
University of Cape Town
Anzio Road, Observatory 7925
Cape Town
South Africa

Refresher Vaccinology Webinar Series for AAVC Alumni

The VACFA group has been granted an unconditional education grant to conduct a refresher vaccinology course via a webinar series. The target audience is alumni of the VACFA's AAVC.

The aim of the refresher is to provide an up-to-date information on the current and emerging trends in vaccinology with a focus on the African continent. The webinar series also provides a forum for networking among the AAVC alumni.

Dates in April 2022: 22nd and 29th
Dates in May 2022: 6th, 20th and 27th

The webinar will start at **12:00 South Africa Standard Time** (SAST; Cape Town GMT+2 hours).
The equivalent times in the different time zones in Africa, Europe, Australia and the USA can be converted using this link: https://www.timeanddate.com/worldclock/converter-classic.html

Programme

The webinars will be presented in English. The format of each webinar is 3-4 keynote speakers followed by a moderated discussion for 50 mins. The last webinar will be on the application of human centred design principles in vaccinology.

**Webinar 1**	Agenda	Time
22nd April 2022	- Welcome<br>- History of the AAVC<br>- Objectives of the webinar series	5 mins
	Immunisation Agenda 2030 with a brief description of the Africa's NIPs situation (including the impacts of Covid-19)	30 mins
	The challenge and solutions to the zero-dose children in Africa, including recent polio outbreak	30 mins
	Africa's vaccines manufacturing capacity: past, present, and the outlook	30 mins
	How can Africa leverage on lessons learnt from Covid-19 pandemic response to strengthen NIPs	30 mins
	The four speakers engage with the participants through the Q&A session	50 mins
	Closure	5 mins
**Webinar 2**		
29th April 2022	- Welcome<br>- Objectives of the webinar series	5 mins
	The basic principles of immunological responses & application of these principles in vaccinology	40 mins
	History and rationale of vaccination schedules	40 mins
	Evidence-based vaccinology in the context of evolving immunization schedules and emergence of new infectious pathogens	40 mins

Vaccines for Africa Initiative
Faculty of Health Sciences
University of Cape Town
Anzio Road, Observatory 7925
Cape Town
South Africa

	Three speakers engage with the participants through a Q&A session	50 mins
	Closure	5 mins
**Webinar 3**		
6th May 2022	Welcome & objectives of the webinar series	5 mins
	Developing vaccines for pandemic preparedness & new vaccines for a safer world- a focus on LMICs	30 mins
	Vaccinology capacity building for Africa's pandemic preparedness- the role of universities and partnerships	30 mins
	Vaccination safety monitoring during Covid-19 pandemic in Africa – Lessons learnt	30 mins
	Three speakers engage with the participants through a Q&A session	50 mins
	Closure	5 mins
**Webinar 4**		
20th May 2022	Welcome & objectives of the webinar series	5 mins
	Global overview of key vaccines pipeline and new technologies	30 mins
	Strategies to improve demand for vaccination and increase vaccines confidence- a focus on Covid-19 vaccination	30 mins
	Digital technology for surveillance, reporting and response to VPDs	30 mins
	Surveillance of VPDS and other infectious diseases - a focus on Africa's capacity during the Covid-19 pandemic	30 mins
	Four speakers engage with the participants through a moderated Q&A session	50 mins
	Closure	5 mins
**Webinar 5**		
27th May 2022	Welcome & objectives of the webinar series	5 mins
	Application of Human-Centred Design (HCD) principles in vaccinology	2 hrs
	Closure, feedback and the next steps	15 mins
