# Supplementary material for: Expanding the reach of vaccinology training in Africa: leveraging the success of the Annual African Vaccinology Course
Source: Front Health Serv. 2023 Sep 1;3:1119858. doi: 10.3389/frhs.2023.1119858 (PMC10505672; doi:10.3389/frhs.2023.1119858)
Supplement: Supplementary file 3 [file Datasheet3.pdf]

# AAVC Post Webinar Series Survey 2022

Thank you for attending VACFA's First Webinar Series!

Greetings from the VACFA's Annual African Vaccinology Course (AAVC) conveners. We have sent you this short questionnaire because you attended the recent AAVC Webinar series and we would appreciate your views on the series in order to improve future webinars.
We have prepared 7 questions that will take you about 5 minutes to complete. Kindly complete and submit by 14 June 2022.
Thank you.
If you have any questions, please contact Dr. Benjamin Kagina (Benjamin.kagina@uct.ac.za) or Dr Nayna Manga (nayna.manga@uct.ac.za)

*Required

1. Email *

2. 1) 1. Which webinars did you attend? (Multiple selection allowed) *

*Tick all that apply.*

- [ ] Webinar #1 (22 April 2022)
- [ ] Webinar #2 (29 April 2022)
- [ ] Webinar #3 (6 May 2022)
- [ ] Webinar #4 (20 May 2022)
- [ ] Webinar #5 (27 May 2022)

3. 2. In your view, to what extent did the webinar series meet the following objective: Provide participants with essential and up to date knowledge on human vaccines? *

*Mark only one oval.*

- ( ) Met the expectations
- ( ) Did not meet the expectations
- ( ) Unsure

4. 3. In your view, to what extent did the webinar series meet the following objective: Refresh & reinforce previous knowledge on Vaccinology *

*Mark only one oval.*

- Met the expectations
- Did not meet the expectations
- Unsure

5. 4. In your view, to what extent did the webinar series meet the following objective: Opportunity for participants to network *

*Mark only one oval.*

- Met the expectations
- Did not meet the expectations
- Unsure

6. 5. In your view, to what extent did the webinar series meet the following objective: Broaden the understanding of the challenges and opportunities in vaccinology at regional and global levels. *

*Mark only one oval.*

- Met the expectations
- Did not meet the expectations
- Unsure

7. 6. Overall evaluation of the webinar series: *

*Mark only one oval.*

- Met the expectations
- Did not meet the expectations
- Unsure

8. 7. Any other feedback that you would like to share with AAVC convenors?

This content is neither created nor endorsed by Google.

Google Forms
